# Mechanistic Insights Underlying Tolerance to Acetic Acid Stress in Vaginal Candida glabrata Clinical Isolates

**DOI:** 10.3389/fmicb.2017.00259

**Published:** 2017-02-28

**Authors:** Diana V. Cunha, Sara B. Salazar, Maria M. Lopes, Nuno P. Mira

**Affiliations:** ^1^Department of Bioengineering, Institute of Bioengineering and Biosciences, Instituto Superior Técnico, Universidade de LisboaLisboa, Portugal; ^2^Faculdade de Farmácia da Universidade de Lisboa, Departamento de Microbiologia e ImunologiaLisboa, Portugal

**Keywords:** acetic acid tolerance, *C. glabrata* stress response, vaginal candidiasis

## Abstract

During colonization of the vaginal tract *Candida glabrata* cells are challenged with the presence of acetic acid at a low pH, specially when dysbiosis occurs. To avoid exclusion from this niche *C. glabrata* cells are expected to evolve efficient adaptive responses to cope with this stress; however, these responses remain largely uncharacterized, especially in vaginal strains. In this work a cohort of 18 vaginal strains and 2 laboratory strains (CBS138 and KUE100) were phenotyped for their tolerance against inhibitory concentrations of acetic acid at pH 4. Despite some heterogeneity has been observed among the vaginal strains tested, in general these strains were considerably more tolerant to acetic acid than the laboratory strains. To tackle the mechanistic insights behind this differential level of tolerance observed, a set of vaginal strains differently tolerant to acetic acid (VG281∼VG49 < VG99 < VG216) and the highly susceptible laboratory strain KUE100 were selected for further studies. When suddenly challenged with acetic acid the more tolerant vaginal strains exhibited a higher activity of the plasma membrane proton pump CgPma1 and a reduced internal accumulation of the acid, these being two essential features to maximize tolerance. Based on the higher level of resistance exhibited by the vaginal strains against the action of a β-1,3-glucanase, it is hypothesized that the reduced internal accumulation of acetic acid inside these strains may originate from them having a different cell wall structure resulting in a reduced porosity to undissociated acetic acid molecules. Both the vaginal and the two laboratory strains were found to consume acetic acid in the presence of glucose indicating that metabolization of the acid is used by *C. glabrata* species as a detoxification mechanism. The results gathered in this study advance the current knowledge on the mechanisms underlying the increased competitiveness of *C. glabrata* in the vaginal tract, a knowledge that can be used to guide more suitable strategies to treat infections caused by this pathogenic yeast.

## Introduction

*Candida glabrata* is a common commensal of the gastrointestinal (GI) and genitourinary (GU) human tracts. Under certain conditions the harmless colonization by *C. glabrata* may progress to more serious infections that can range from mild mucosal infections to large disseminated mycoses in which this yeast crosses the bloodstream and may colonize any major organ. While invasive candidiasis is known to occur essentially in susceptible populations such as immunocompromised patients or the elderly, mucosal infections caused by *Candida* spp. are very frequent even among the healthy population. Indeed, vulvovaginal candidiasis (VVC), the most frequent form of superficial candidiasis, has been estimated to affect 75% of all women during their life (Sobel et al., 1998; Saporiti et al., 2001; Sobel, 2016). In more serious cases, the vulvovaginal infections caused by *Candida* spp. can occur in a recurrent manner this being a condition known as recurrent vulvovaginal candidiasis (RVVC). It is estimated that each year approximately 138 million of women suffer from RVVC worldwide (Sobel, 2016). Due to their high rates of morbidity and recurrence, VVC and RVVC are among the infectious diseases posing a more serious social-economical burden for healthcare systems (Sobel, 2016). Although *Candida albicans* is the more relevant causative agent of VVC and RVVC, it is clear that the incidence of infections caused by non-*C. albicans* *Candida* species, and in particular, by *C. glabrata*, is increasing (Fidel et al., 1999; Achkar and Fries, 2010; Zhang et al., 2014; Sobel, 2016). This increase is considered specially dangerous because *C. glabrata* is highly resilient to fluconazole and also to other azoles used in the treatment of VVC and RVVC (Sobel et al., 1998, 2001; Achkar and Fries, 2010; Danby et al., 2012; Zhang et al., 2014).

To thrive in the vaginal tract *C. glabrata* cells have to cope with a number of stresses including, among others, alterations in the external pH, fluctuations in concentration of nutrients, presence of different hormones, the activity of the host immune system and a competing microbiota. Thorough metagenomic surveys undertaken with different female populations worldwide revealed that although some variation in the isolated species and in their-rank abundance is observed, the vaginal microbiome is essentially composed by lactic acid bacteria (Zhou et al., 2010; Ravel et al., 2011; Chaban et al., 2014). This conservation has been associated with the ability of lactic acid bacteria to produce organic acids that in the acidic niche of the vaginal tract can exert a toxic effect for pathogens (Morales and Hogan, 2010; Hickey et al., 2012; Aldunate et al., 2015; Sobel, 2016). Consistent with this protective effect attributed to commensal vaginal bacteria, the use of large-spectrum anbitiotics is a known-risk factor for the development of candidiasis (Huang et al., 2011; Ben-Ami et al., 2012; Sobel, 2016). Furthermore, several recent studies have demonstrated the ability of different Lactobacilii to *in vitro* inhibit growth and virulence of *C. albicans* (Gil et al., 2010; Parolin et al., 2015). Interestingly, the vaginal microbiomes of women with RVVC appear to be less enriched in lactic acid bacteria (Yan et al., 2009; Shipitsyna et al., 2013).

Lactic acid is the organic acid found in highest abundance in vaginal fluid, this being attributable to the activity of homolactic bacteria (Boskey et al., 1999, 2001). However, lactic acid bacteria also carry out heterolactic fermentation, which besides producing lactic acid also produces acetic acid and ethanol. Consistently, acetic acid has also been found to be present in the vaginal fluid in concentrations ranging between 1 and 4 mM (Owen and Katz, 1999; Boskey et al., 2001). However, when an overgrowth of bacteria occurs (a condition known as bacterial vaginosis) the concentration of acetic acid increases prominently reaching approximately 120 mM (Chaudry et al., 2004). The acidic pH of the vaginal tract (within the range 3.6–4.5) (Boskey et al., 1999) potentiates the toxic effect exerted by acetic and lactic acids since a substantial percentage of the acid molecules will exist in their undissociated form (RCOOH), which has a well-described antimicrobial effect (Mira et al., 2010b). For example at pH 4, 80% of the acetic acid (pKa of 4.76) molecules will be undissociated while the percentage of undissociated lactic acid (pKa of 3.86) will be of 42%. To succeed in the colonization of the vaginal tract and to avoid exclusion from this niche in favor of co-colonizing microbes, *Candida* cells are expected to have evolved dedicated adaptive responses allowing them to tolerate the presence of acetic and lactic acids at low pH.

The molecular mechanisms underlying the adaptive responses of *C. glabrata* to high concentrations of lactic and acetic acid under low pH conditions are still scarce but extensive knowledge has been obtained in *Saccharomyces cerevisiae* (reviewed in Mira et al., 2010b), a species closely related to *C. glabrata*. Due to its lipophilicity undissociated acetic acid/lactic acid molecules are able to freely permeate the plasma membrane of yeast cells dissociating directly in the near-neutral cytosol. Consistent with this, a drop in internal pH has been reported to occur when *C. glabrata* cells are challenged with inhibitory concentrations of acetic acid (Bairwa and Kaur, 2011; Ullah et al., 2013b). To counter-act the acid-induced intracellular acidification *C. glabrata* cells rely on the activity of the plasma membrane H^+^-ATPase CgPma1 which pumps the exceeding protons to the outside of the cell, this response also contributing to maintain the electrochemical gradient across the plasma membrane which is essential for the activity of secondary transporters (Ullah et al., 2013b; Bernardo et al., 2017). Because acetate and lactate cannot cross the plasma membrane by passive diffusion due to their negative charge, their accumulation inside *C. glabrata* is expected. It was recently shown the important role of the multidrug resistance transporter CgTpo3 in contributing to reduce the internal concentration of acetic acid when cells are subjected to inhibitory concentration of this acid (Bernardo et al., 2017). CgAqr1, also recently characterized as a drug efflux pump, was also found to provide protection against acetic acid in *C. glabrata*, however, in this case this did not resulted from a role of this transporter in mediating the extrusion of the acid anion (Costa et al., 2013).

In this work we have examined tolerance to acetic acid stress at pH 4, a pH physiologically relevant in the vaginal tract, of a cohort of *C. glabrata* vaginal strains and two laboratory strains (CBS138 and KUE100). Afterward, based on the results of the phenotypic screening performed, a smaller set of strains found to be differently tolerant to acetic acid was selected for further studies aiming to obtain mechanistic insights underlying the differential levels of tolerance observed.

## Materials and Methods

### Strains and Growth Media

Two *C. glabrata* reference strains were used in this work: CBS138 (also named as ATCC2001), recovered from the intestinal tract, and the KUE100 strain, derived from ATCC2001 and used as a laboratory strain for gene manipulation (Ueno et al., 2011). Besides these reference strains, 18 clinical vaginal isolates recovered during epidemiological surveys undertaken in hospitals of the Lisbon area (Lopes et al., 2006, 2007) were also used. All strains were batch-cultured at 30°C, with orbital agitation (250 rpm), in liquid minimal media MM which contains, per liter, 1.70 g yeast nitrogen base (YNB) without amino acids and ammonium (Difco Laboratories, Detroit, MI, USA), 20 g glucose (Merck Millipore, Darmstadt, Germany) and 2.65 g (NH_4_)_2_SO_4_ (Merck Millipore). When required the pH of this growth medium was adjusted to 4.0 or to 6.4 using HCl or NaOH. Solid media was obtained by supplementing the corresponding liquid growth medium with 20 g per liter of agar (Iberagar). If required, the adjustment of solid media pH was made after agar supplementation to pH 4.5 or pH 6.4 before autoclaving the media.

### Comparison of Susceptibility to Acetic Acid and to Other Environmental Stressors Based on Spot Assays

For the spot assays cell suspensions of the different strains were batch-cultured in MM liquid medium (adjusted at pH 4.0 or 6.4) at 30°C with orbital agitation (250 rpm) until mid-exponential phase (OD_600nm_ 0.8–1.0 ± 0.05) and then diluted to a standardized OD_600nm_ of 0.05 ± 0.005 in 1 ml of sterilized-deionized water. Two subsequent dilutions (1:5 and of 1:25) of the initial cell suspension were prepared and then applied as spots (4 μl) onto the surface of agarized MM plates supplemented with the different concentrations of acetic acid. After inoculation, the agar plates were incubated at 30°C for 1–2 days, depending on the severity of growth inhibition. To have a quantitative analysis of the results obtained each spot density was estimated using ImageJ software and the results obtained were compiled in a matrix used to build heat-maps. This same experimental setup was used to compare the susceptibility of the different strains to H_2_O_2_, to calcofluor white and to Congo red. The stock solution of acetic acid used to supplement the growth medium was prepared in sterile water and adjusted at pH 4.5 with NaOH.

### Comparison of Susceptibility to Acetic Acid in Liquid Growth Medium

To complement the results obtained with the spot assays, growth of the two laboratory strains (KUE100 and CBS138) and of the vaginal strains was compared after 24 h of cultivation in 96-multiwell plates containing MM growth medium (at pH 4) or this same medium supplemented with 60 or 80 mM of acetic acid. For this the cells of the different strains were cultivated in MM growth medium until mid-exponential phase and then re-inoculated (at an OD_600nm_ of 0.05) in fresh medium either or not supplemented with the acid. The microplates were incubated at 30°C with 250 rpm agitation and after 24 h the OD_600nm_ was measured.

### Growth Curves in the Presence of Acetic Acid in Liquid Growth Medium

Growth in 96-multiwell plates of the vaginal isolates VG281, VG216, VG99, and VG49 and of the laboratory strains KUE100 and CBS138 in liquid MM growth medium (at pH 4) supplemented with inhibitory concentrations of acetic acid was followed along 55 h. For this, cells of the different strains were cultivated in liquid MM medium (at pH 4.0) until mid-exponential phase (OD_600nm_ between 0.8 ± 0.05 and 1.0 ± 0.05) and then diluted (in deionized sterile water) to prepare 5 mL of a cell suspension having an initial OD_600nm_ of 0.1 ± 0.001. 100 μL of these diluted cell suspensions were used to re-inoculate the 96-well plates containing 100 μL of fresh MM growth medium (2x concentrated) (at pH 4.0) supplemented with acetic acid in different concentrations ranging from 60 to 100 mM. Growth of each strain was followed based on the increase of OD_600nm_.

### [1-^14^C]-Acetic Acid Accumulation Assays

The accumulation ratio of [1-^14^C]-acetic acid (defined as the ratio between the intracellular and extracellular concentrations of radiolabeled acetic acid) was compared in the laboratory strain KUE100 and in the clinical isolates VG281, VG216, and VG99. Cells of the different strains were cultivated in liquid MM medium (at pH 4.0) at 30°C with orbital agitation (250 rpm) until mid-exponential phase (OD_600nm_ = 0.8 ± 0.05), harvested by filtration, washed one time with fresh medium and finally resuspended in 5 ml of this same medium to obtain a dense cell suspension (OD_600nm_ = 0.7 ± 0.05). The cell suspensions were incubated for 5 min at 30°C in a water bath with orbital agitation (150 rev/min). After this time, 21 μM of radiolabeled [1-^14^C]-acetic acid (sodium salt from GE Healthcare, Piscataway, NJ, USA; 9.25 MBq) and 60 mM of cold acetic acid were added to the cell suspension. Culture samples were taken after 1, 5, 10, 15, 20, 25, and 30 min of incubation. To measure extracellular [1-^14^C]-acetic acid, a 100 μL culture sample was collected and the supernatant was recovered by centrifugation in a tabletop centrifuge (12000 rpm, 1 min). For quantification of intracellular [1-^14^C]-acetic acid, 200 μL of culture sample were filtered through pre-wetted glass microfiber filters (Whatman GF/C) and washed with cold water. The radioactivity was measured in a Beckman LS 5000TD scintillation counter. Non-specific adsorption of acetic acid to the filters and to the cells was assessed and taken into consideration (less than 5% of the total bound-radioactivity). Internal cell volume (V_i_) of *C. glabrata*, used to calculate the internal concentration of acetic acid, was considered constant and equal to 2.5 μL (mg dry weight)^-1^ (Costa et al., 2013).

### *In vivo* Estimation of *C. glabrata* PM-H^+^-ATPase Activity

The *in vivo* activity of *C. glabrata* PM-H^+^-ATPase was estimated based on the ability of cells to acidify the extracellular medium (Bairwa and Kaur, 2011; Bernardo et al., 2017). The proton pumping activity was compared in KUE100, VG281, VG216, and VG99 strains. For this cells were cultivated until mid-exponential phase in liquid MM medium (at pH 4.0), harvested by filtration, washed twice with distilled water and incubated at 30°C in sorbitol solution (20 g/L, pH 4.0) for 30 min to deactivate the plasma membrane PM-H^+^-ATPase. After this time, cells were centrifuged, washed with water (at pH 4.0) to remove any sorbitol residue and finally resuspended in distilled water (at pH 4.0) to obtain a dense cell suspension (OD_600nm_ ∼ 20.0 ± 2.0). Each assessment of *C. glabrata* PM-H^+^-ATPase activity was conducted in a water-jacketed cell (capacity 5 ml), at 30°C, with agitation, by adding 3.0 mL of water (at pH 4.0) and 1 mL of cell suspension. The stock solution of acetic acid used (3 M) was adjusted to pH 4.0 using HCl as the acidulant. After mixing, pH of the suspension was rapidly adjusted to 4.0 ± 0.1 using HCl or NaOH. Activation of *C. glabrata* PM-H^+^-ATPase in each assay was initiated by the addition of 1 ml of glucose 100 g/L (at pH 4) to the mixture (yielding a final glucose concentration in the reaction mixture of 2%). pH of the mixture was measured every 10 s by potentiometry using a pH microelectrode (6.0202.100, Metrohm) attached to a Metrohm 691 pH meter.

### Quantification of Acetic Acid and Glucose in Culture Supernatants

The concentration of acetic acid and glucose present in the supernatant of cultures growing in MM growth medium (either or not supplemented with acetic acid) was accompanied by HPLC. For this, KUE100, VG281, VG216, and VG99 cells were cultivated, for about 60 h, in liquid MM medium either or not supplemented with 60 mM acetic acid (at pH 4.0). Samples of culture supernatants were taken at appropriate time intervals and then separated in an Aminex HPX-87H column, eluted at room temperature with 0.005 M H_2_SO_4_ at a flow-rate of 0.6 ml/min, during 30 min. A refractive-index (RI) detector was used to quantify glucose while acetic acid was detected using an UV-Vis detector set at 210 nm. Under the conditions used glucose and acetic acid had retention times of 9.2 and 14.3 min, respectively. Reproducibility and linearity of the method were tested and concentrations of the compounds were estimated based on appropriate calibration curves.

### Susceptibility to Lyticase

To assess susceptibility of the laboratory strain KUE100 and of the vaginal isolates VG281, VG99, VG49, and VG216 to lyticase a previously described protocol was used (Simoes et al., 2006; Costa et al., 2015). Briefly, the cells were cultivated in MM growth medium (at pH 4) until mid-exponential phase, harvested by filtration and inoculated in 30 mL of 0.1 mM sodium phosphate buffer at pH 7.0. Afterward, 10 μg/ml of lyticase from *Arthrobacter luteus* were added to the cell suspensions and cell lysis was taken as the decrease in OD_600nm_ registered every 20 min.

## Results

### Tolerance of *C. glabrata* Vaginal Isolates to Acetic Acid at a Low pH

**FIGURE 1:**
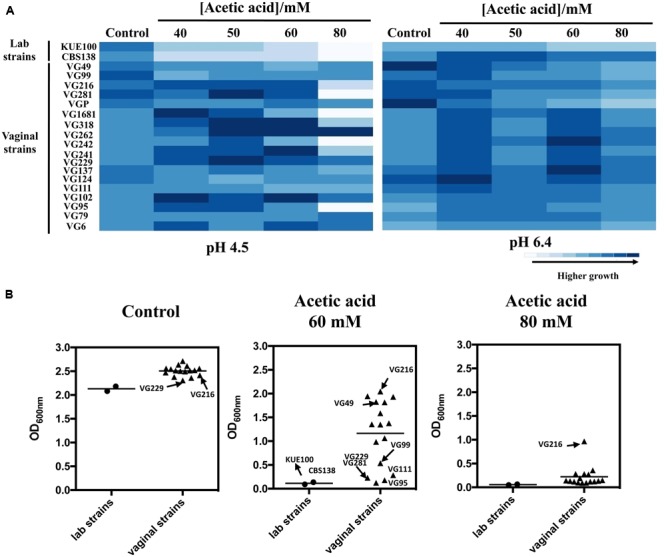
**Comparison of the susceptibility to acetic acid of laboratory, vaginal and intestinal *Candida glabrata* vaginal strains.** Susceptibility to acetic acid of the two laboratory strains KUE100/CBS138 and of the vaginal isolates was compared by spot assays **(A)** and during cultivation in liquid growth medium **(B)**. In the case of the spot assays, the strains were cultivated in agarized MM growth medium (at pH 4.5 or at pH 6.4) either or not supplemented with different concentrations of acetic acid. After 3 days of incubation at 30°C, pictures were taken and the cell density of each spot measured using the software ImageJ. The results shown here are representative of, at least, three independent experiments that gave essentially the same pattern of growth. For the comparison of growth in liquid medium, the strains were cultivated in 96-wells microplates for 24 h in MM growth medium (at pH 4) either or not supplemented with 60 or 80 mM acetic acid. After 24 h of growth the OD_600nm_ of all the cultures was measured. The results obtained are means of at least five different experiments.

To successfully colonize the vaginal tract (pH ∼4) *C. glabrata* cells are hypothesized to have evolved specific adaptive responses to cope with the presence of acetic acid at a low pH. To test this hypothesis we have compared the susceptibility to inhibitory concentrations of acetic acid of 18 vaginal isolates with the tolerance of two largely used laboratory strains, CBS138, retrieved from the gastrointestinal tract, and KUE100, derived from CBS138. Within the cohort of isolates tested only VG99 and VG95 were recovered from patients with medically diagnosed vaginal candidiasis, while the others were recovered from asymptomatic women. Tolerance of the vaginal isolates and of the two laboratory strains to acetic acid was first compared in solid MM medium at pH 4.5 and at pH 6.4 (**Figure 1A**), these pHs being representative of the range of variations known to occur in vaginal pH during the menstrual cycle (Boskey et al., 1999). For all the strains the inhibitory effect of acetic acid was much more pronounced at pH 4.5 (below acetic acid p*Ka*) than at pH 6.4 (above acetic acid p*Ka*), consistent with the idea that the undissociated form of the acid (CH_3_COOH) is the one exerting toxicity against *C. glabrata*. A significant heterogeneity concerning tolerance to acetic acid was observed among the set of vaginal isolates tested, with some isolates being largely tolerant to acetic acid (e.g., VG216) while others were found to be more susceptible (e.g., VG281, VG95, VG111; **Figure 1A**). Despite this heterogeneity the majority of the vaginal strains tested was clearly more tolerant to acetic acid than the two laboratory strains used (**Figure 1A**). To obtain a more quantitative view on the differential levels of tolerance to acetic acid exhibited by the vaginal strains and the laboratory strains, we have compared their growth after 24 h of cultivation in liquid MM growth medium supplemented with acetic acid (60 and 80 mM; **Figure 1B**). The results obtained largely confirmed the results obtained in the spot assays, with strain VG216 clearly emerging as a highly acetic acid-tolerant isolate and the two laboratory strains being the more susceptible strains (**Figure 1B**). Some differences were observed in fitness of the vaginal strains even in the absence of acetic acid, however, the isolates found to be more tolerant to acetic acid did not coincided with those exhibiting higher growth in control conditions (**Figures 1A,B**). It was also noticeable a lower fitness of the laboratory strains, in comparison with the vaginal strains, in the absence of acetic acid during growth in MM growth medium (**Figures 1A,B**).

**FIGURE 2:**
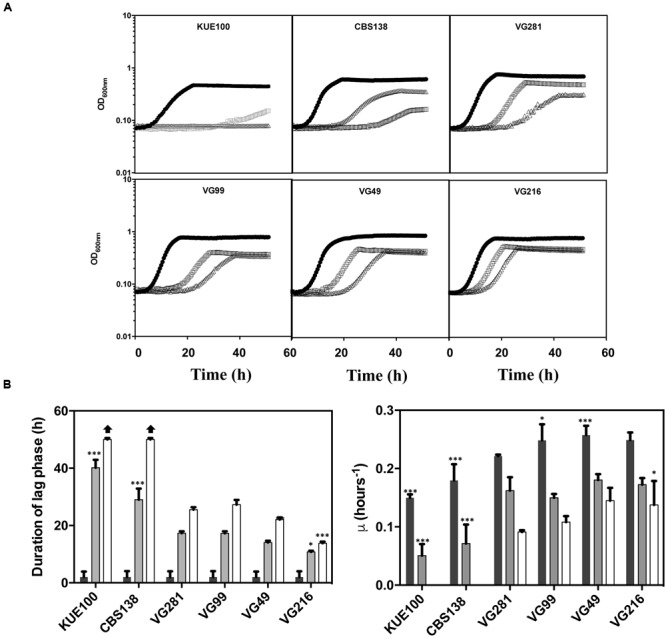
**Comparison of the growth curves of the tolerant *C. glabrata* vaginal isolates VG281, VG216, VG99, and VG49 and of the susceptible laboratory strains KUE100 and CBS138 in the presence of acetic acid. (A)** Growth curve of the vaginal isolates and of the laboratory strains was compared in MM growth medium (at pH 4) (■ dark bars) or in this same growth medium supplemented with 60 mM (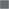 gray bars) or 80 mM (△ white bars) of acetic acid. Growth of the different cultures was based on the increase of OD_600nm_ of the culture. The growth curves shown are representative of at least five independent experiments that resulted in a similar growth pattern. **(B)** Based on the growth curves shown in **(A)**, the duration of the lag phase and the maximum growth rate of the different strains during cultivation in the presence or absence of acetic acid were calculated. The results shown are the average of the results obtained for the different replicas of the growth curves that were obtained for each strain in each condition. To assess the differences obtained for the strains statistical analysis of the data was performed using as a reference the VG281 strain. ^∗∗∗^*p*-value below 0.001; *^∗∗^p*-value below 0.01; ^∗^*p*-value below 0.05.

To obtain more detailed information regarding the effect of acetic acid in the growth inhibition of the vaginal and of the laboratory strains, the growth curve in liquid MM medium (at pH 4), either or not supplemented with acetic acid, of a selected set of vaginal isolates differently tolerant to acetic acid – VG281∼VG99 < VG49 < VG216- **Figure 1B** – and of the highly susceptible laboratory strains KUE100 and CBS138, was accompanied for about 55 h (**Figure 2**). As described above, during cultivation in unsupplemented MM medium all the vaginal strains tested exhibited a growth rate higher than the one registered for the two laboratory strains (**Figure 2**). Cultivation of the different unadapted populations in the presence of 60 mM acetic acid induced a lag phase period that was of approximately 9 h for the more tolerant isolates VG49 and VG216, 11 h for the intermediately tolerant strains VG281 and VG99 and 30 and 40 h for the highly susceptible strains CBS138 and KUE100 (**Figure 2**). In the presence of 80 mM acetic acid the lag phase duration increased for all the strains, this being particularly evident for the more susceptible strains CBS138, KUE100 and VG281 (**Figure 2**). Remarkably, in the highly tolerant isolate VG216 the increase in the lag phase period was almost negligible (**Figure 2**). In what concerns to the growth rate of adapted cells exponentially growing in the presence of acetic acid the sole differences of notice were the lower growth rate of the two laboratory strains, in comparison with the vaginal strains, and the higher growth rate of VG216 in the presence of 80 mM acetic acid (**Figure 2**). On the overall the differences registered in the growth rate of adapted cells of the vaginal strains were much less pronounced than those registered for the duration of the lag phase period, sustaining the idea that the differential levels of tolerance to acetic acid of the strains are essentially the result of different adaptation rates at which they adapt after sudden exposure to inhibitory concentrations of the acid.

### Tolerance of *C. glabrata* Vaginal Strains to Other Environmental Stressors

**FIGURE 3:**
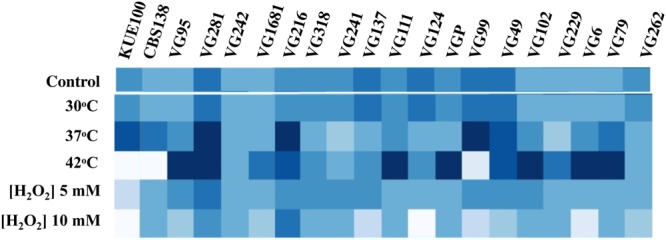
**Comparison of the susceptibility of the laboratory and of the vaginal *C. glabrata* strains to heat stress and to H_2_O_2_.** Susceptibility of the vaginal isolates and of the laboratory strains KUE100 and CBS138 to H_2_O_2_ and to heat stress compared by spot assays. To test susceptibility to H_2_O_2_ the strains were cultivated in MM growth medium (at pH 4.5) either or not supplemented with inhibitory concentrations of this chemical. After 3 days of incubation at 30°C pictures of the plates were taken and the cell density of each spot measured using the software ImageJ to reconstruct the heat map shown. Comparison of the susceptibility of the strains to heat stress was undertaken in the same conditions with the difference that in the latter case after inoculation the strains were incubated at 37 or 42°C.

To see if the higher tolerance to acetic acid of the vaginal strains was linked to a generalized tolerance to stress their susceptibility to other environmental stressors, including exposure to heat stress and to inhibitory concentrations of H_2_O_2_, was tested (**Figure 3**). As observed for acetic acid, the isolates exhibited different levels of tolerance against heat or H_2_O_2_ with some strains being highly resilient to heat stress but tolerant to H_2_O_2_ (e.g., VG111) and vice-versa (e.g., VG99). Most significant is the fact that the vaginal strains found to be more tolerant to acetic acid did not coincided with those exhibiting higher tolerance to H_2_O_2_ or to heat stress (**Figure 3**). For example, VG281 was among the isolates found to be more tolerant to H_2_O_2_ or to heat stress but was only mildly tolerant to acetic acid (**Figures 1**–**3**). This observation shows that increased tolerance to acetic acid does not appear to result from a generalized resilience of the strains to environmental stress but it rather seems to result from the more tolerant strains having evolved dedicated adaptive responses.

### Acetic Acid-Tolerant and -Susceptible *C. glabrata* Strains Co-consume Glucose and Acetic Acid

**FIGURE 4:**
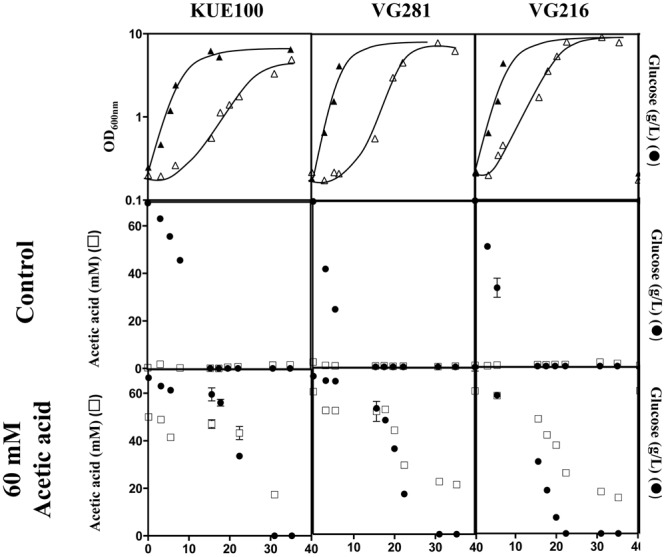
***Candida glabrata* strains are able to consume acetic acid in the presence of glucose.** The laboratory strain KUE100 and the vaginal strains VG281 and VG216 were cultivated in liquid MM medium (at pH 4.0) until mid-exponential phase and were then re-inoculated in fresh medium (at pH 4) either or not supplemented with 60 mM acetic acid. Growth was followed during approximately 40 h during which samples of culture supernatants were harvested and used for the quantification of acetic acid (□) and glucose (●) concentrations by HPLC, as detailed in Section “Materials and Methods.” The results shown are means of those obtained in three independent experiments that gave essentially the same results.

*Candida glabrata* has been shown to assimilate acetate when this is used as the sole carbon source (Ueno et al., 2011; Mota et al., 2015). Given this, we have examined whether the *C. glabrata* strains used in our study could consume acetic acid in the presence of glucose, as this could function as a mean to detoxify the presence of high concentrations of acetic acid. To test this hypothesis, the consumption of glucose and acetic acid was followed in cultures of the VG281 and VG216 vaginal isolates and of the laboratory strain KUE100 along cultivation in MM growth medium (at pH 4 and containing 2% glucose) supplemented or not with 60 mM acetic acid (**Figure 4** and results not shown). In the absence of the acid, all the strains rapidly exhausted the glucose available in the growth medium, although the rate of consumption of the vaginal strains was above the one exhibited by the laboratory strain KUE100 (**Figure 4**). Supplementation of the growth medium with 60 mM acetic acid (at pH 4) arrested glucose consumption in the more susceptible strains KUE100 and VG281 but this was resumed when cells adapted and entered the exponential phase of growth (**Figure 4**). In the more tolerant isolate VG216; the supplementation of the medium with acetic acid did not arrest the consumption of glucose, although it slightly reduced its rate (**Figure 4**). In all cases the amount of acetic acid present in the growth medium decreased concomitantly with the decrease observed in the concentration of glucose indicating co-consumption of the two carbon sources (**Figure 4**). We could also confirm that the intermediately tolerant vaginal strains VG49 and VG99 and the susceptible strain CBS138 also consume acetic acid in the presence of glucose (results not shown). On the overall these results suggest that co-utilization of glucose and acetate is generalized within the *C. glabrata* species, this likely representing an important mean by which cells detoxify the presence of high concentrations of acetic acid. Nevertheless, this alone could not assure the differential level of tolerance exhibited by the strains against acetic acid since it was observed to occur both in susceptible and in tolerant strains.

### Acetic-Acid Tolerant Vaginal *C. glabrata* Isolates Accumulate Less Acid Intracellularly and Show Increased Resistance to Lyticase

**FIGURE 5:**
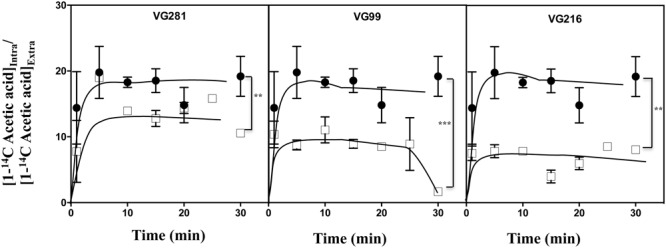
**Acetic acid-tolerant vaginal isolates accumulate less acid intracelularly than susceptible strains Time-course representation of the accumulation ratio of [1-^14^C]-acetic acid in vaginal isolates VG281 (moderately tolerant to acetic acid), VG216 and VG99 (tolerant to acetic acid), in comparison with the susceptible laboratory strain KUE100 (●), during cultivation in MM (at pH 4.0) supplemented with 60 mM of cold acetic acid.** The results obtained were representative of, at least, five independent experiments. ^∗∗^*p*-value below 0.01; ^∗∗∗^*p*-value below 0.001.

**FIGURE 6:**
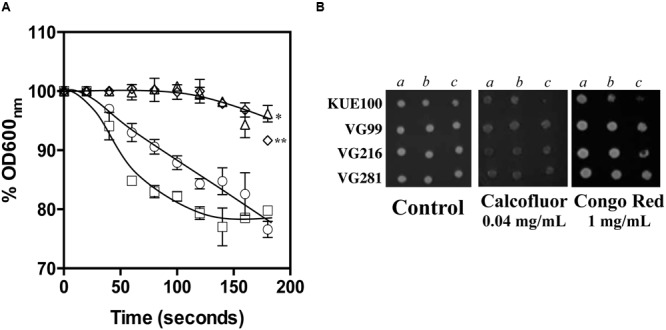
**Comparison of *C. glabrata* laboratory strain KUE100 and of the differently acetic acid tolerant vaginal clinical isolates to lyticase (A)**, calcofluor and Congo Red **(B)**. **(A)** To test susceptibility of the different strains to lyticase these were cultivated in unsupplemented liquid MM medium until mid-exponential phase at standardized OD_600nm_ of 0.8 ± 0.005 and then re-inoculated into 0.1 mM sodium phosphate buffer (pH 7.0) supplemented with 10 μg/ml lyticase from *Arthrobacter luteus*. The results obtained were representative of, at least, three independent experiments. Statistical analysis of the data was performed using the last time point and using the KUE100 strain as a reference ^∗^*p*-value below 0.05; ^∗∗^*p*-value below 0.01. **(B)** To test susceptibility of the strains to inhibitory concentrations of calcofluor white or Congo Red the strains were cultivated, at 30°C for 3 days, in agarised MM medium supplemented with the indicated concentrations of the two cell wall perturbing agents. The pictures shown are representative of the results obtained in three independent experiments that were performed.

The reduction of the internal accumulation of acetic acid is one of the mechanisms contributing for increased tolerance to this weak acid in *S. cerevisiae* (Mira et al., 2010b) and this may also be beneficial for *C. glabrata* cells. Thus, the levels of acetic acid accumulated inside the vaginal isolates VG281, VG216, and VG99 after sudden exposure to an inhibitory concentration of the acid were compared with those attained in the susceptible laboratory strain KUE100. For this, unadapted cells of the different strains cultivated in MM growth medium (at pH 4) were suddenly exposed to a cold concentration of acetic acid (60 mM) together with a non-toxic concentration of radiolabeled acetic acid. The accumulation of radiolabeled acid (taken as the ratio between the extracellular and intracellular concentrations) was followed during the first 30 min of incubation in the presence of cold and radiolabeled acetic acid molecules (**Figure 5**). The results obtained show that all the vaginal strains accumulate less radiolabeled acetic acid than the laboratory strain KUE100, this being particularly prominent in the highly tolerant isolate VG216 (**Figure 5**). Since these assays were undertaken using non-adapted cells and the lower accumulation of acetic acid in the vaginal strains VG216 and VG99 was observed after only 1 min of incubation in the presence of the acid, we have hypothesized whether these strains could be *a priori* less permeable to the acid. Several studies undertaken in *S. cerevisiae* have associated the reduction in permeability to organic acids to a modification of cell wall structure, which could result in a different porosity to the undissociated acid molecules (Simoes et al., 2006; Ullah et al., 2013a). In this context we have compared resistance of the vaginal strains VG281, VG99, VG216 and of the reference strain KUE100 to the activity of the β-1,3-glucanase lyticase, this being a simple assay that can be used to monitor alterations in cell wall structure under stress in yeasts, including in *C. glabrata* (Simoes et al., 2006; Costa et al., 2015). The acetic acid-tolerant isolates VG216 and VG99 were highly resistant to the activity of lyticase while the laboratory strain KUE100 was markedly sensitive (**Figure 6A**). Strain VG281, which accumulated more acetic acid than strains VG99 and VG216 but less than the KUE100 strain, showed an intermediate level of resistance against lyticase (**Figure 6A**). Consistent with the idea that the cell wall of the vaginal strains and of the laboratory strain KUE100 differs, this latter strain was also found to be more susceptible to inhibitory concentrations of calcofluor white and Congo red, two known cell wall damaging agents (**Figure 6B**).

### Upon Acetic Acid Challenge the Activity of the Plasma Membrane Proton Pump H^+^-ATPase is Higher in the Acid-Tolerant Vaginal *C. glabrata* Isolates

**FIGURE 7:**
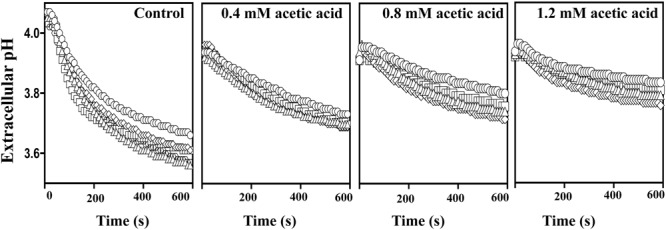
**External medium acidification promoted by CgPma1 H^+^-ATPase in laboratory strain KUE100 and in the differently acetic acid-tolerant vaginal clinical isolates.** Cell suspensions of the different strains – KUE100 (□), VG216 (Δ), VG99 (♢), and VG281 (◌) – were cultivated in MM growth medium (at pH 4) until mid-exponential phase and then de-energized for 30 min, as detailed in Section “Materials and Methods.” After this time concentrated cell suspensions were energized with a pulse of glucose to assess the proton pumping capacity prompted by CgPma1 H^+^-ATPase activity, in the presence or absence of acetic acid. The results obtained are means of at least four independent experiments that gave, essentially, the same results.

Exposure to acetic acid stress leads to intracellular acidification and this is counter-acted by the activation of the plasma membrane H^+^-ATPase CgPma1 (Bairwa and Kaur, 2011; Ullah et al., 2013b). Taking this into account we have compared the activity of CgPma1 in the highly acetic acid-tolerant vaginal isolate VG216, in the intermediately tolerant isolates VG49, VG99, and VG281 and in the highly susceptible strain KUE100 (**Figure 7**). The activity of CgPma1 was measured based on the capacity of the different strains to acidify the external environment upon supplementation with glucose and/or acetic acid, a method used before with success to monitor proton pumping activity in acetic acid-stressed *S. cerevisiae* and *C. glabrata* cells (Mira et al., 2010a; Bairwa and Kaur, 2011). In the absence of acetic acid the proton pumping activity of all the vaginal isolates tested was clearly above the one exhibited by KUE100 cells (**Figure 7**). Supplementation of the cell suspensions with acetic acid led, in all cases, to a reduction in the activity of the proton pump, this being attributed to the passive diffusion of the acid into the cell interior that leads to ATP depletion and dissipates the electrochemical gradient maintained across the plasma membrane, two essential features for maximal CgPma1 activity (**Figure 7**). Despite this, the acid-induced inhibition of CgPma1 was more evident for the more susceptible strains KUE100 and VG281 than for the more tolerant isolates VG49, VG99, and VG216 (**Figure 7**).

## Discussion

The molecular mechanisms underlying the ability of *C. glabrata* to thrive in the presence of acetic acid at low pH are still scarcely understood, although it is known that this is an important trait to assure competitiveness of this pathogen in the vaginal tract, in particular when dysbiosis occurs and the concentration of this organic acid increases drastically (Chaudry et al., 2004). In this work we have gathered mechanistic insights into tolerance to acetic acid at low pH in a set of *C. glabrata* vaginal strains. Higher tolerance to acetic acid could not be associated with a generalized resilience of the strains to environmental stress since vaginal strains found to be moderately tolerant to acetic acid were highly tolerant to heat stress or to H_2_O_2_ and vice-versa. The strains tested in our study were retrieved from different women and therefore their phenotypic heterogeneity concerning tolerance to different stressors is likely to result from them having a different genetic background, eventually driven by different host-dependent selective pressures. However, the existence of phenotypic diversity concerning multiple stressors should be advantageous even for *C. glabrata* vaginal populations that colonize a single individual since this increases competitiveness and provides versatility to overcome multiple environmental insults. As such, the occurrence of phenotypic diversity among *C. glabrata* clinical isolates, including among isolates collected from the same patient, has been described concerning resistance to antifungals, adhesion, or cell hydrophobicity (Shin et al., 2007; Ahmad et al., 2014).

Despite the heterogeneity the vaginal strains were, in general, more tolerant to acetic acid at a low pH than the two laboratory strains CBS138 and KUE100 tested. This was first hypothesized to have resulted from these lab strains having an intestinal origin where the pressure to selective acetic acid-tolerant strains would be smaller since the pH in this niche is higher than the one registered in the vaginal tract. However, we could not confirm this hypothesis since we have also phenotyped 10 gastrointestinal isolates and found several isolates exhibiting high levels of tolerance to acetic acid (results not shown). Thus with the data available we cannot say if the increased tolerance to acetic acid at a low pH is exclusive of vaginal isolates or if it may also be observed in isolates collected from other niches. The extensive utilization of the CBS138 and KUE100 strains in the laboratory may have led to some domestication thereby rendering these strains more susceptible to environmental stressors than wild-type isolates. This hypothesis is further supported by the herein observed lower growth rates exhibited by the two laboratory strains even in the absence of stress. A similar domestication phenomenon has been described to occur in *S. cerevisiae* lab strains with consequences in tolerance to several stress agents (Lewis et al., 2010). A comparative genomic analysis between the herein studied vaginal isolates, the CBS138 strain and eventually other gastrointestinal isolates, will help clarify this matter. Nevertheless, the comparative analysis herein performed clearly shows distinct phenotypic traits between the vaginal strains and the two laboratory strains, some of them with impact in mediating tolerance to acetic acid such as the activity of the CgPma1 proton pump and the reduced levels of acetic acid accumulated intracelularly (discussed below).

Both the vaginal and the laboratory strains tested were found to be able to consume acetic acid when glucose was present in the growth medium thus indicating that *C. glabrata* detoxifies the presence of high concentrations of acetic acid by metabolizing it. In line with this, a recent microarray analysis of *C. glabrata* KUE100 cells growing in a growth medium supplemented with glucose and acetic acid (in comparison with cells growing in a medium containing solely glucose) revealed a prominent up-regulation of genes encoding enzymes involved in acetate metabolism including isocitrate lyase *CgICL1* (ORF CAGL0J03058g, up-regulated ∼3-fold), malate-synthase *CgMLS1* (ORF CAGL0L03982g, up-regulated ∼2-fold) and *CgACS1*, encoding acetyl-CoA synthase (ORF CAGL0L00649g, up-regulated ∼2-fold) (Bernardo et al., 2017). The highly acetic acid-tolerant species *Zygosaccharomyces bailii* has also been reported to co-consume acetic acid and glucose and this was suggested to contribute for its increased resilience against this acid (Sousa et al., 1998; Guerreiro et al., 2012; Rodrigues et al., 2012). In *S. cerevisiae* this mechanism is blocked because glucose exerts a potent repressive effect over metabolism of acetate, thereby resulting in increased susceptibility of this species to acetic acid (Sousa et al., 1998; Guerreiro et al., 2012). Recently a set of clinical *S. cerevisiae* strains having the glucose repressive effect alleviated were identified (Childers et al., 2016) this being attributed to the need of human colonizing strains to be metabolically flexible to cope with the nutrient deprivation that characterizes the different infection sites (Childers et al., 2016). Consistently, in *C. albicans* the presence of glucose was also found not to affect the ability of this yeast species to metabolize other carbon sources (Sandai et al., 2012; Childers et al., 2016). The results obtained in our study clearly show that acetate metabolism is not repressed by glucose suggesting that glucose repression is also alleviated in *C. glabrata*. The vaginal fluid contains about 0.5% of glucose in addition to lactic acid, acetic acid, glycogen, and other components that may also serve as potential carbon sources (Owen and Katz, 1999; Childers et al., 2016). In this sense, the alleviation of glucose repression should contribute to favor *C. glabrata* colonization of the vaginal niche contributing for enhanced metabolic versatility. However, this ability to consume acetic acid even when glucose is present in the environment does not alone explain the differential tolerance of the strains to acetic acid since it was observed to occur at similar rate both in the susceptible strains KUE100, CBS138, and VG281 and in the highly tolerant strain VG216. The ability to counter-act the intracellular acidification imposed by acetic acid is likely to play a detrimental role in determining the ability of the strains to trigger metabolization of the acid, since *C. glabrata* metabolic enzymes required for glucose and acetate catabolism may have a reduced activity when the internal pH decreases, as demonstrated to occur in *S. cerevisiae* (Holyoak et al., 1996). In this context it is interesting to note that the highly acetic acid-tolerant strain VG216, that resumed consumption of acetic acid and glucose much earlier than the more susceptible strains, was the strain exhibiting higher levels of CgPma1 activity upon acetic acid challenge. A better control of the internal pH has been found to determine tolerance to acetic acid at a single cell level in *S. cerevisiae* and in *Z. bailii* (Stratford et al., 2014; Fernandez-Nino et al., 2015) and it is likely to play also a detrimental role in determining tolerance in *C. glabrata*.

Another important factor that should contribute for a more robust tolerance to acetic acid of the vaginal strains is the reduced internal accumulation of acetic acid that was observed upon sudden exposure to inhibitory concentrations of the acid, in comparison with the high levels that are attained in the highly susceptible strain KUE100. The reorganization of *S. cerevisiae* wall structure under acetic acid stress has been pointed out as an important adaptive response as it may reduce porosity to undissociated acid molecules thus lowering the rate at which the acid enters the cells (Simoes et al., 2006; Ullah et al., 2013a). In this sense it is interesting that the highly lyticase-resistant isolate VG216 accumulated very low levels of acetic acid, while the lyticase-susceptible strains KUE100 and VG281 accumulated higher levels of acetic acid. The cell wall structure of *C. glabrata* has been described to be under a strong selective pressure during infection of the human host (as reviewed by Hall, 2015). It is thus possible that during colonization of the vaginal tract significant alterations in the cell wall architecture may occur, some of these contributing to increase tolerance to acetic acid at a low pH.

*Candida glabrata* is an important vaginal colonizer and this in part due to its remarkable ability to cope with multiple environmental stresses, which include the presence of high concentrations acetic acid at a low pH. In this work mechanistic insights underlying tolerance to acetic acid in a cohort of vaginal *C. glabrata* strains were gathered. In general the vaginal strains were found to be more tolerant to acetic acid than the two laboratory strains KUE100 and CBS138, confirming the hypothesized idea that adaptation to the vaginal environment involves the development of adaptive responses increasing tolerance to this organic acid. Metabolization of acetic acid even when glucose is present in the growth medium appears to be used as a detoxification mechanism employed by *C. glabrata*, however, this alone does not render cells tolerant to acetic acid. Our results show that the vaginal strains exhibit a higher activity of the plasma membrane proton pump CgPma1 and a reduced internal accumulation of the acid, these being two essential features to increase acid tolerance. On the overall the results presented in this study provide a contribution to further understand the adaptive responses evolved by *C. glabrata* to improve its competitiveness in the vaginal tract, a knowledge that can be incorporated in the design of treatments of infections caused by this pathogenic yeast.

## Author Contributions

Under the supervision of NPM, which coordinated and conceived the study, DC and SBS performed all the experiments concerning tolerance of the isolates to the different stressors, measurement of levels of intracellular accumulation of acetic acid, estimation of CgPma1 activity and quantification of glucose and acetic acid consumption by HPLC. MML collected the isolates and contributed in their identification.

## Conflict of Interest Statement

The authors declare that the research was conducted in the absence of any commercial or financial relationships that could be construed as a potential conflict of interest.
